# Generation of functional CD8+ T Cells by human dendritic cells expressing glypican-3 epitopes

**DOI:** 10.1186/1756-9966-29-48

**Published:** 2010-05-13

**Authors:** James O'Beirne, Farzin Farzaneh, Phillip M Harrison

**Affiliations:** 1 Department of Liver Studies & Transplantation, Kings College London, Denmark Hill Campus, Bessemer Road, London, SE5 9RS, UK; 2 Department of Haematological Medicine, Kings College London, The Rayne Institute, Coldharbour Lane, London, SE5 9NU, UK; 3 The Royal free Sheila Sherlock Liver Centre and Department of Surgery, Royal Free Hospital, Pond Street, Belsize Park, London NW3, UK

## Abstract

**Background:**

Glypican 3 (GPC-3) is an oncofoetal protein that is expressed in most hepatocellular carcinomas (HCC). Since it is a potential target for T cell immunotherapy, we investigated the generation of functional, GPC-3 specific T cells from peripheral blood mononuclear cells (PBMC).

**Methods:**

Dendritic cells (DC) were derived from adherent PBMC cultured at 37°C for 7 days in X-Vivo, 1% autologous plasma, and 800 u/ml GM-CSF plus 500 u/ml IL-4. Immature DC were transfected with 20 μg of *in vitro *synthesised GPC-3 mRNA by electroporation using the Easy-ject plus system (Equibio, UK) (300 V, 150 μF and 4 ms pulse time), or pulsed with peptide, and subsequently matured with lipopolysaccharide (LPS). Six predicted GPC-3 peptide epitopes were synthesized using standard f-moc technology and tested for their binding affinity to HLA-A2.1 molecules using the cell line T2.

**Results:**

DC transfected with GPC-3 mRNA but not control DC demonstrated strong intracellular staining for GPC-3 and *in vitro *generated interferon-gamma expressing T cells from autologous PBMC harvested from normal subjects. One peptide, GPC-3_522-530 _FLAELAYDL, fulfilled our criteria as a naturally processed, HLA-A2-restricted cytotoxic T lymphocyte (CTL) epitope: i) it showed high affinity binding to HLA-A2, in T2 cell binding assay; ii) it was generated by the MHC class I processing pathway in DC transfected with GPC-3 mRNA, and iii) HLA-A2 positive DC loaded with the peptide stimulated proliferation in autologous T cells and generated CTL that lysed HLA-A2 and GPC-3 positive target cells.

**Conclusions:**

These findings demonstrate that electroporation of GPC-3 mRNA is an efficient method to load human monocyte-derived DC with antigen because *in vitro *they generated GPC-3-reactive T cells that were functional, as shown by interferon-gamma production. Furthermore, this study identified a novel naturally processed, HLA-A2-restricted CTL epitope, GPC-3_522-530 _FLAELAYDL, which can be used to monitor HLA-A2-restricted CTL responses in patients with HCC. Further studies are required to investigate whether anti-GPC-3 immunotherapy has a role in the treatment of GPC-3 dependent tumours, such as HCC.

## Background

Increasing evidence suggests that immune responses play an important role in the control of cancer and manipulation of the immune system to recognize and attack cancer cells may be a valuable form of therapy [1]. Hepatocellular carcinoma (HCC), which is the third most common cause of cancer death world-wide [2], is a potential target for immunotherapy [3] because there are numerous documented cases of spontaneous regression [4] and the presence of cytotoxic tumour infiltrating lymphocytes (TIL) at histological examination is associated with a better prognosis after liver resection or transplantation [5]. Infusion of T lymphocytes, activated with anti CD3 and interleukin 2 (IL2), improved disease-free survival after HCC resection, suggesting a role for T cell immunotherapy in this setting [6]. However, current methods of isolation and *in vitro *expansion of T lymphocytes are cumbersome and expensive, and the durability of any anti-tumour immune response induced by administration of non-antigen specific, *in vitro *expanded T cells is unknown [7].

Many tumours, including HCC, express tumour-associated antigens (TAA) that might serve as potential targets for antigen-specific T cell immunotherapy. Glypican 3 (GPC-3), a 580 amino acid glycosylphosphatidylinositol-linked heparan sulphate proteoglycan, is expressed in foetal liver and plays an important role in foetal development because it facilitates the interaction of growth factors with their cognizant receptors [8]. It is rarely detected in adult liver but is reactivated in 72% of HCC [9], where its expression is correlated with a poor prognosis [10]. Intradermal vaccination of BALB/c mice with a GPC-3 peptide (EYILSLEEL), restricted to the murine MHC-I molecule H-2K^d^, mixed with incomplete Freund's adjuvant induced epitope specific, cytotoxic T lymphocytes (CTL) [11] and immunization using dendritic cells (DC) pulsed with this peptide prevented the growth of GPC-3 positive tumours [12]. Mice vaccinated with DC expressing GPC-3 as a transgene were also found to have protective immunity against subsequent challenge with GPC-3 positive melanoma cells [13]. In a study of 20 HCC patients treated with locoregional therapy, 16 (80%) were found to have TAA-specific CD8+ T cells, including T cells directed against GPC-3 [14]. Furthermore, the magnitude of the TAA-specific CD8+ T-cell response was a significant independent prognostic factor for tumour-free survival. These data suggest that GPC-3 is a novel HCC-associated antigen but further studies are required to investigate the immunogenicity of human GPC-3 and to establish any therapeutic potential.

In this study, we demonstrate that human monocyte-derived DC expressing GPC-3 antigen are able to induce, *in vitro*, GPC-3-reactive T cells that are functional, as shown interferon-gamma production. We also describe a novel naturally processed, immunogenic epitope, GPC-3_522-530 _FLAELAYDL, which is restricted to HLA-A2, a common class 1 allele in various ethnic groups, including Asians and Caucasians.

## Methods

### Cell lines

T2 cells (HLA-A*0201) and the human hepatocellular carcinoma cell line HepG2 (HLA-A*0201 and GPC-3 positive) were obtained from ATCC and expression of HLA-A2 and GPC-3 confirmed in the latter using flow cytometry, after staining with monoclonal antibodies against HLA-A2.1 (BB7.2, Dako, UK), and GPC-3 (Biomosaics Inc, Burlington, USA), respectively (data not shown). The cell lines were cultured in RPMI (Gibco, UK) or DMEM (Cambrex, UK), respectively, supplemented with 10% foetal calf serum (FCS) (Cambrex, UK) and antibiotics (penicillin G 100 IU/ml and Streptomycin 50 μg/ml).

### T2 binding assays

The prediction tools SYFPEITHI [15] and HLAmotif [16] were used to reveal GPC-3 peptide epitopes with predicted strong binding to HLA-A2. The top 30 peptides were reassessed using RankPep [17], which also predicts epitopes generated by the proteasome, and 6 peptide epitopes were selected (Table 1). These peptides were synthesized using standard f-moc technology (>95% purity, as determined by reverse phase HPLC; Sigma, UK), along with an AFP-derived, HLA-A2-binding peptide (GVALQTMKQ) [18], and a random, non-HLA-A2 binding, control peptide (RGYVYQGL). The AFP peptide has only one anchor but is an "immunodominant" epitope [19] and its use was convenient because T cells reactive to this epitope have been shown to lyse HepG2 cells. Due to the hydrophobicity of peptides binding to HLA-A2, the lyophilized peptides were resuspended in DMSO at 10 mM.

**Table 1:** GPC-3 peptides predicted to bind to HLA-A2 and be processed by the proteasome, and control peptides used in the study

GPC-3 peptide	Position	Sequence
1	229-237	FLQALNLGI
2	522-530	FLAELAYDL
3	299-307	YILSLEELV
4	186-194	GLPSALDI
5	222-230	SLQVTRIFL
6	169-177	ELFDSLFPV
AFP peptide		GVALQTMKQ
Control peptide		RGYVYQGL

The selected epitopes were tested for their binding affinity to HLA-A2.1 molecules using the cell line T2, which is deficient in TAP1 and TAP 2 (transporters associated with antigen processing 1 and 2) [20]. Although T2 cells express very low levels of HLA-A2.1 molecules under normal culture conditions, cell surface expression is upregulated when appropriate peptides bind and stabilize the HLA-A2.1 molecule. Thus, up-regulation of HLA-A2.1 expression in T2 cells by a peptide is regarded as an indication of it being an HLA-A2.1-restricted epitope [19]. HLA-A2.1 expression on the T2 cell surface was quantified by staining the cells with HLA-A2-specific antibody (1 μg/ml), as described [21]. To assess the stability of the HLA-A2-peptide complex, 1 × 10^5 ^T2 cells were incubated for 3 hours at 37°C with various concentrations of peptide, 5 nM β_2 _microglobulin and 5 μg/ml Brefeldin A, which inhibits the transportation of protein through the Golgi and hence blocks delivery of new MHC class I molecules to the cell surface. Cells were washed at the end of each time point and stained for cell surface HLA-A2 expression and then analyzed by flow cytometry.

### Construction of RNA expression vector and in vitro transcription of mRNA

**Figure 1:**
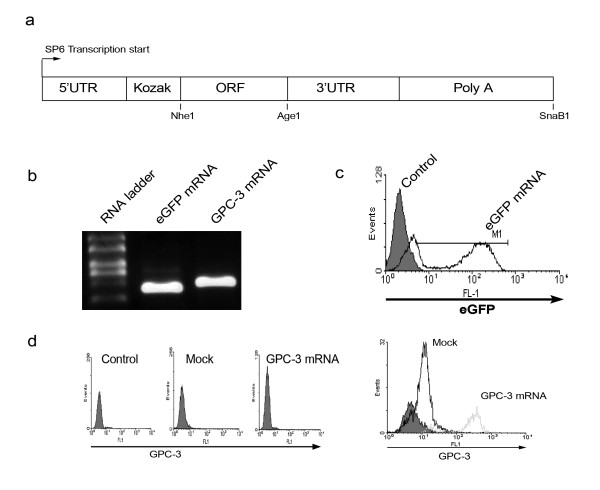
**Expression of transgenes in monocyte-derived dendritic cells following transfection by electroporation of mRNA**. **a**. Diagram of expression vector between SP6 transcription initiation site and SnaB1 restriction enzyme site. **b**. Denaturing agarose gel showing *in vitro *transcribed eGFP and GPC-3 mRNA. **c**. eGFP expression in monocyte-derived DC as determined by flow cytometry, 20 hours after mock transfection (*filled area*) or transfection with 20 μg eGFP mRNA (*open area*), when 64% of DC were positive for eGFP. **d**. GPC-3 expression in matured, monocyte-derived DC as determined by flow cytometry after staining with anti-GPC-3 antibody; *left panel *shows cell surface expression of GPC-3, 20 hours after mock transfection or transfection with 20 μg GPC-3 mRNA, staining with isotype control antibody is shown for comparison; *right panel *shows intracellular expression after DC were permeabilised by drop-wise addition of the cells to ice cold 70% ethanol, 20 hours after mock transfection (*thick line*) or transfection with 20 μg GPC-3 mRNA (*pale line*), staining with isotype control antibody is shown for comparison (*filled area*).

An RNA expression vector was constructed on the backbone of the PGEM 5Z(+) vector (Promega, Southampton, UK). A 76 nucleotide poly-A sequence was cloned into the vector between the Sph1 and Apa1 sites and a sequence containing a new multiple cloning cassette and the 3' untranslated region of the human α-globin gene, to increase the intracellular stability of mRNA transcripts [22], was inserted between the Sac1 and Sph1 sites. Subsequently, the cDNA sequences encoding the open reading frames of either GPC-3 or enhanced green fluorescent protein (eGFP) were inserted between Nhe1 and Age1 sites in the new cloning cassette, downstream of the SP6 promoter site of PGEM5Z and upstream of the mRNA stabilizing sequences (Figure 1a). The 1.74 kb GPC-3 open reading frame sequence was generated by PCR from reverse transcribed RNA extracted from HepG2 cells using primers 5'-CGAGCTAGCATGGGCCGGGACCGTG and 5'-AGGACCGGTGTGCACCAGGAAGAAGAAGC, which incorporated restriction sites for Nhe1 and Age1, respectively. The vector was sequenced to confirm authenticity.

The vector was linearized using a SnaB1 restriction site, which is immediately downstream of the poly A sequence, and the resulting linear DNA was isolated by gel extraction. Following the manufacturers instructions, the linear DNA (1 μg) served as template in an SP6 mMessage Machine reaction (Ambion, Huntingdon, UK). After 3 hours at 37°C the capped mRNA was extracted and purified using RNAeasy columns (Qiagen, Crawley, UK). Transcripts were then analyzed and quantified by denaturing agarose gel electrophoresis before use.

### Dendritic cell culture and mRNA transfection

Fresh heparinised, peripheral blood samples were obtained from HLA-A2 positive, normal subjects, according to a protocol approved by The Kings College Hospital Ethical Committee (LREC Protocol number 01/248). Informed, written consent was obtained and the study was performed according to the principles of World Medical Association Declaration of Helsinki. DC were derived from PBMC essentially as described by Romani et al [23]. Adherent cells (7 × 10^6 ^per well of 6-well plates; Nunc, UK) were cultured at 37°C for 7 days in X-Vivo, 1% autologous plasma, and 800 u/ml GM-CSF plus 500 u/ml interleukin-4 (IL-4) (both from R&D Systems, Abingdon, UK) with cytokine replenishment after 3 days.

Immature DC were transfected with mRNA by electroporation in 400 μL of X-Vivo with no supplements in a 4 mm cuvette using the Easy-ject plus system (Equibio, Ashford, UK) at 300 V and 150 μF and a pulse time of 4 ms. After electroporation, DC were resuspended in 3 ml of X-Vivo with antibiotics and rested for 1 hour.

### ELISPOT and T cell proliferation assays

PBMC were depleted of HLA class II positive cells, using anti-HLA Class II-coated magnetic particles (Dynabeads, Dynal Biotech, Wirral, UK). ELISPOT assay (U-Cytech, Netherlands) was performed to determine the number of cells producing interferon-gamma. Briefly, HLA class II-depleted cells were seeded in 96 well plates (1 × 10^5^/well) and co-cultured with autologous, γ-irradiated (4,000 rads), matured DC (1 × 10^4^/well) in serum-free X-Vivo medium supplemented on days 1, 3 and 7 of culture with IL-2 (20 U/ml) and IL-7 (10 ng/ml) (both from R&D systems, UK). Cells were re-stimulated after 7 days with autologous, γ-irradiated, matured DC (1 × 10^4^/well) in the presence of IL-2 and IL-7 and 24 hours after the second stimulation with antigen-loaded DC, T cells were washed and plated at 1 × 10^5 ^cells/well of the ELISPOT plates, which were incubated for 5 hours before being washed and developed. T cells supplemented with PHA (10 μg/ml) acted as a positive control.

To assess T cell proliferation, HLA class II-depleted cells were seeded in 96 well plates (1 × 10^5^/well) and co-cultured with autologous, γ-irradiated (4,000 rads), matured DC (1 × 10^4^/well) in serum-free X-Vivo medium supplemented on days 1, 3 and 7 of culture with IL-2 (20 U/ml) and IL-7 (10 ng/ml) (both from R&D systems, UK). Cells were re-stimulated after 7 days with autologous, γ-irradiated, matured DC (1 × 10^4^/well) in the presence of IL-2 and IL-7 and cultures incubated for a further 5 days; ^3^H-thymidine (Amersham Pharmacia Biotech, Amersham, Bucks, UK) was added for the last 18 hours of culture. DC were either transfected with mRNA or pulsed with 1 μM peptides for 3 hours, and matured with LPS (100 ng/ml) (Sigma, UK) for 16 hours.

### Chromium release assay

A chromium release assay was used to assess the ability of CTL to lyse target cells. Briefly, PBMC were enriched for CD8+ cells by depletion of CD4+ cells using anti-CD4 microbeads (MACS beads, Miltenyi Biotec, Bergisch Gladbach, Germany) and these cells (1 × 10^6 ^cells/well) were co-cultured with autologous, γ-irradiated (4,000 rads) DC (1 × 10^5 ^cells/well in 6 well plates), which had been pulsed with 1 μM peptides for 3 hours and matured with LPS (100 ng/ml) for 16 hours. Cells were cultured in serum-free X-Vivo medium supplemented on days 1, 3 and 7 with IL-2 (20 U/ml) and IL-7 (10 ng/ml) (both from R&D systems, UK). Cells were re-stimulated after 7 days with peptide-pulsed DC and, 5 days after the second stimulation, the cytotoxic activity of the expanded T cells was measured by chromium release assay. Target cells (HepG2) were labelled with 200 μCi Na_2_^51^CrO_4 _(Amersham, UK) in 0.5 ml DMEM containing 10% FCS for 60 minutes at 37°C. The cells were washed 3 times with warm medium and plated at 5 × 10^3 ^cells/well in round-bottomed 96 well plates (Nunc). Effector cells were added at graded effector/target ratios in a final volume of 200 μl/well. After 4 hours incubation at 37°C, 50 μl of the culture supernatant was harvested and radioactivity counted in a scintillation counter (Beckmann, USA). For controls, maximum chromium release was achieved by the addition of 10% Triton-X and spontaneous release was assessed with medium alone. Percentage specific lysis was calculated as (Experimental release - spontaneous release)/(Maximum release - spontaneous release) × 100. All determinations were made in triplicate.

### Statistical analysis

All statistical analysis was performed using the Statistical Program for Social Sciences (SPSS 14.0 for Windows; SPSS Inc., Chicago, Illinois, USA), using the Mann-Whitney test for unpaired and the Wilcoxon Signed Ranks test for paired data. A difference between two variables was considered significant when the two-tailed *P *value was < 0.05.

## Results

### Expression of transgenes in monocyte-derived dendritic cells following electroporation of mRNA

The yield of each SP6 mMessage Machine reaction was around 20 μg of capped mRNA from 1 μg of linear DNA template. Transcripts were extracted using RNAeasy columns and the quality of the mRNA confirmed by denaturing agarose gel electrophoresis (Figure 1b). Electroporation of 20 μg eGFP mRNA into monocyte-derived DC resulted in 64% of DC expressing eGFP at 20 hours after transfection, as assessed by FACS analysis (Figure 1c). Monocyte-derived DC transfected with 20 μg GPC-3 mRNA and matured with LPS were stained with anti GPC-3 antibody (1 μg/ml) and analyzed by flow cytometry but cell surface expression of GPC-3 could not be detected (Figure 1d left panel) until DC were permeabilised, by drop-wise addition of the cells to ice cold 70% ethanol (Figure 1d right panel). These findings demonstrate that transfection of DC with the synthetic mRNA resulted in high levels of expression of GPC-3 or the control protein, eGFP.

### T cells generated by DC transfected with GPC-3 mRNA are functional *in vitro*

**Figure 2:**
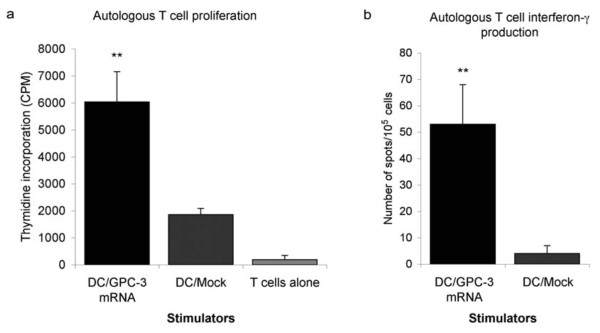
**T cells generated by DC transfected with GPC-3 mRNA are functional *in vitro***. PBMC were depleted of HLA class II positive cells and co-cultured with autologous, γ-irradiated, LPS matured DC in serum-free X-Vivo medium supplemented on days 1, 3 and 7 of culture with IL-2 (20 U/ml) and IL-7 (10 ng/ml). After 7 days, T cells were re-stimulated with the same DC for a further 5 days. **a**. T cell proliferation (1 × 10^5^/well) was measured by ^3^H-thymidine incorporation, T cells were cultured alone, with DC (1 × 10^4^/well) transfected with 20 μg GPC-3 mRNA, or mock transfected DC. **b**. ELISPOT assay for interferon-γ production was performed on T cells (1 × 10^5^/well) stimulated by DC transfected with 20 μg GPC-3 mRNA or mock transfected DC.

GPC-3 mRNA transfected DC but not mock transfected DC induced proliferation of autologous T cells (Figure 2a), indicating that T cells reacting to GPC-3 epitopes are represented in the peripheral T cell repertoire. ELISPOT assay for interferon-gamma production found that DC expressing GPC-3 generated significantly more T cells producing interferon-gamma than mock transfected DC (53 ± 15 versus 4 ± 3 spots per well, respectively; p < 0.01) (Figure 2b). These data demonstrate that monocyte-derived DC transfected with GPC-3 mRNA and matured with LPS were able to process and present GPC-3 derived epitopes, resulting in the proliferation of autologous T cells, which were functional as assessed by interferon-gamma production.

### Assessment of binding affinity of GPC-3 peptides to HLA-A2

**Figure 3:**
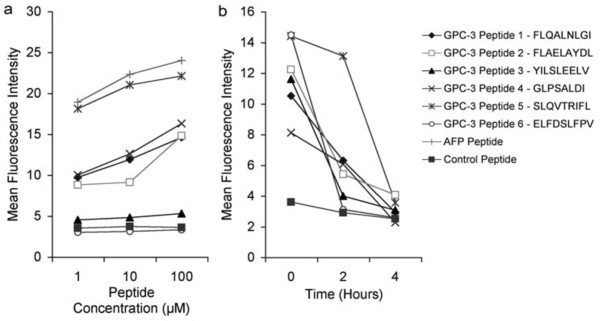
**Binding affinity of GPC-3 peptides to HLA-A2**. T2 cells were plated into 96-well U-bottomed plates at 1 × 10^5 ^cells per well in 200 μL X-Vivo (Biowhittaker) and cultured overnight at 18°C to increase cell surface HLA-A2 expression. **a**. 3 hours after pulsing with increasing concentration of GPC-3 peptides, positive control (AFP) peptide or negative control (random) peptide plus 5 nM β_2 _microglobulin and incubation at 37°C, T2 cells were stained with a FITC-conjugated HLA-A2 specific antibody and examined by flow cytometry; **b**. T2 cells were stained with a FITC-conjugated HLA-A2 specific antibody and examined by flow cytometry at time points after the cells had been incubated for 3 hours at 37°C with 100 μM peptide, 5 nM β_2 _microglobulin and 5 μg/ml Brefeldin A. The data shown are representative of three independent experiments.

Among the 6 GPC-3 peptides tested, peptides 1, 2, 4 and 5 (GPC-3 229-237, 522-530, 186-194 and 222-230, respectively) showed significant binding affinities, whereas peptides 3 and 6 (GPC-3 299-307 and 169-177, respectively) did not show significant binding under the conditions used in these experiments (Figure 3). However, none of the GPC-3 peptides exhibited very strong binding to HLA-A2, as all demonstrated weaker binding than the "immunodominant" AFP peptide (GVALQTMKQ).

### Induction of functional T cells *in vitro *by GPC-3 peptide-loaded DC

**Figure 4:**
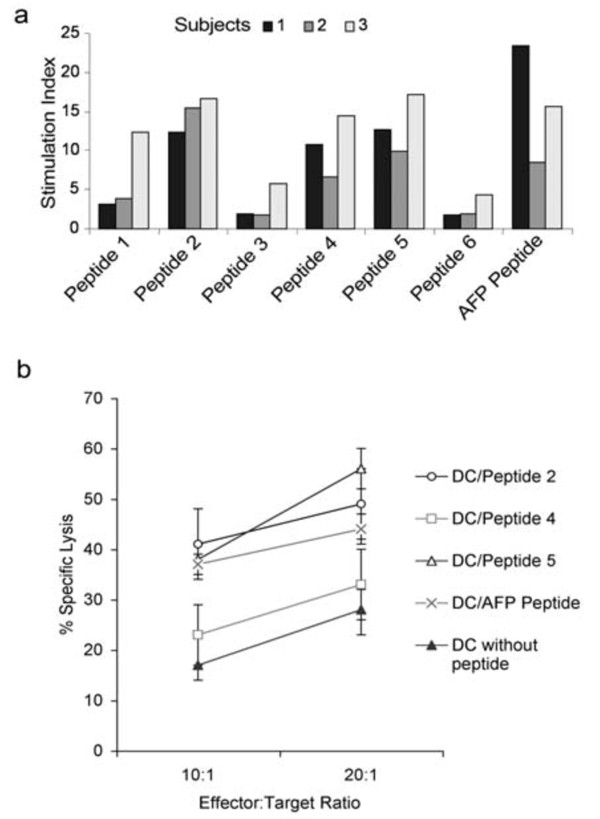
**Induction of functional T cells *in vitro *by GPC-3 peptide-loaded DC**. **a**. PBMC (1 × 10^5^/well), depleted of HLA class II positive cells, from 3 healthy HLA-A2 positive subjects were stimulated twice with autologous, monocyte-derived DC (1 × 10^4^/well), which had been pulsed with 1 μM peptides for 3 hours, matured with LPS and γ-irradiated, in serum-free X-Vivo medium supplemented with IL-2 (20 U/ml) and IL-7 (10 ng/ml). T cell proliferation was measured by ^3^H-thymidine incorporation, Stimulation Index is ratio of T cell proliferation due to peptide-pulsed DC ÷ control DC. **b**. CD8+ enriched T cells were stimulated twice by autologous, γ-irradiated, peptide-pulsed, matured DC. The ability of these CD8+ T cells to lyse HepG2 cells was assessed by chromium release assay. Target cells (HepG2) were labelled with 200 μCi Na_2_^51^CrO_4 _and plated (5 × 10^3 ^cells/well) in round-bottomed 96 well plates. Effector cells were added at graded effector/target ratios and after 4 hours incubation at 37°C, 50 μl of the culture supernatant was harvested and radioactivity counted in a scintillation counter. Spontaneous release was <15% in all assays. *Error bars *reflect standard error of mean of 3 experiments.

Since the binding of a peptide to MHC class I molecules does not necessarily mean that the epitope will be able to induce MHC-restricted CTL, we examined whether these peptides could generate peptide-specific T cells from normal subjects. Peptide 2 GPC-3_522-530 _FLAELAYDL, peptide 4 GPC-3_186-194 _GLPDSALDI, and peptide 5 GPC-3_222-230 _SLQVTRIFL were presented by HLA-A2, inducing T cell proliferation, as assessed by thymidine incorporation, in all donors to a level similar to that induced by DC loaded with the "immunodominant" AFP peptide (Figure 4a). Although, peptide 1 had shown high affinity binding to HLA-A2, only 1 out of the 3 subjects had highly reactive T cell proliferation to this epitope. DC loaded with peptides 3 and 6 were unable to stimulate autologous T cell responses in 2 subjects and induced only low level T cell proliferation in the other. These data showed a good correlation between the peptide's observed binding affinity for HLA-A2 and the ability of DC loaded with peptide to induce autologous T cell proliferation.

T cell function was assessed by their ability to lyse chromium-labelled HepG2 cells (HLA-A2+, GPC-3+) as targets. CD8+ enriched T cells were stimulated twice by autologous, γ-irradiated, peptide-pulsed, matured DC. T cells harvested after two rounds of stimulation with DC pulsed with GPC-3 peptides 2 or 5, or the "immunodominant" AFP peptide efficiently lysed HepG2 cell targets (Figure 4b). Notably, although T cells were generated by DC loaded with GPC-3 peptide 4, GPC-3_186-194 _GLPDSALDI, they were not significantly better at lysing targets than T cells stimulated by control, unpulsed DC. This finding suggests that either CTL reacting against this epitope (GPC3_186-194 _GLPDSALDI) were ineffective or this epitope was not generated by the proteasome in HepG2 cells and hence not presented in association with HLA-A2 at the cell surface. There were insufficient CD8+ T cells generated against epitope GPC3_186-194 _GLPDSALDI to test whether they could lyse targets pulsed with GLPDSALDI peptide.

### Processing of HLA-A2-restricted GPC-3 epitopes by mRNA transfected DC

**Figure 5:**
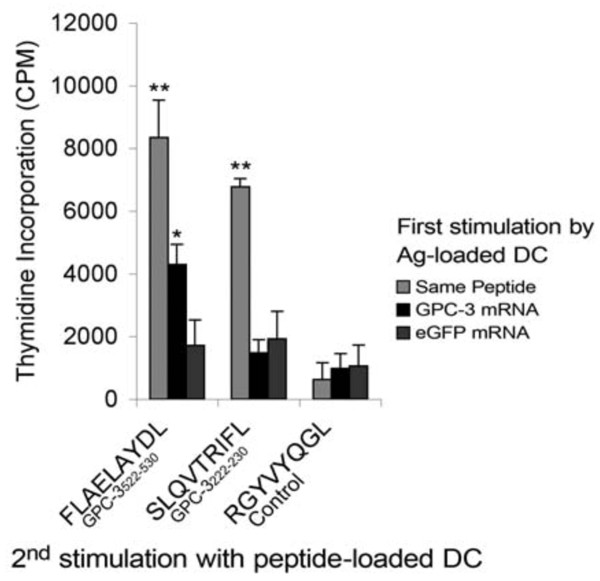
**Processing of HLA-A2-restricted GPC-3 epitopes by mRNA transfected DC**. T cell pools were expanded firstly by a round of stimulation with autologous, irradiated, matured DC pulsed with 1 μM GPC-3 or irrelevant control peptides, or DC transfected with either GPC-3 mRNA or eGFP mRNA as control, followed by a second round of stimulation with autologous, irradiated, matured DC pulsed with 1 μM GPC-3 or irrelevant control peptides. T cell proliferation was assessed by thymidine incorporation, at a stimulator to responder ratio of 1:10. * p < 0.05 and ** p < 0.01 compared to T cells stimulated in the first round by eGFP mRNA transfected DC; *error bars *reflect standard error of mean of 3 experiments.

On the basis of the above results, GPC-3 peptide epitopes 2 and 5 were selected for further investigation to establish whether these epitopes are generated and presented in association with HLA-A2 by DC transfected with GPC-3 mRNA. T cell pools were generated by stimulation of PBMC with autologous, irradiated, matured DC pulsed with 1 μM GPC-3 or irrelevant control peptides, or with autologous, irradiated, matured DC transfected with GPC-3 mRNA or eGFP mRNA, as control. A second round of stimulation was performed with autologous, irradiated, matured DC pulsed with 1 μM GPC-3 or irrelevant control peptides. DC pulsed with peptide 2 (GPC-3_522-530 _FLAELAYDL) not only induced proliferation in T cells previously expanded by DC pulsed with the same peptide, as expected, but also in T cells previously expanded by DC transfected with GPC-3 mRNA but not eGFP mRNA, indicating that the GPC-3 mRNA transfected DC expressed HLA-A2/FLAELAYDL complex on the cell surface and were able to expand viable CD8+ T cell precursors. Hence, the GPC-3_522-530 _FLAELAYDL epitope is generated by the MHC class I processing pathway in DC. In contrast, although DC pulsed with peptide 5 (GPC-3_222-230 _SLQVTRIFL) induced proliferation in T cells previously expanded by DC pulsed with the same peptide, they failed to stimulate proliferation of T cells previously expanded by DC transfected with either GPC-3 mRNA or eGFP mRNA, suggesting that the epitope, SLQVTRIFL, was not processed for presentation in association with HLA-A2 in the GPC-3 mRNA transfected DC (Figure 5).

## Discussion

In this study, we show that T cells reacting to GPC-3 epitopes are represented in the peripheral T cell repertoire of normal human subjects. Despite being exposed to this oncofoetal protein during embryonic development not all GPC-3-specific T cells were deleted during the ontogeny of the immune system. The data clearly demonstrate that monocyte-derived DC electroporated with GPC-3 mRNA efficiently presented GPC-3 epitopes to autologous T cells and generated functional GPC-3-reactive T cells *in vitro *as shown by production of interferon-gamma.

Several studies have confirmed the very high sensitivity and specificity of GPC-3 over-expression for differentiating HCC from non-malignant liver tissue [9,24-28]. Nonetheless, a recent study reported GPC-3 immunoreactivity in inflammatory liver biopsies from patients with chronic hepatitis C [29] and a further study reported the up-regulation of GPC-3 in monocyte-derived DC after maturation [30]. The discovery of GPC-3 protein in non-malignant adult tissue, whether inflamed liver or mature DC, challenges the hypothesis that GPC-3 is a potential target TAA for HCC immunotherapy because of the spectre that the generation of GPC-3-reactive T cells would induce auto-immune disease. Reassuringly, in the present study, flow cytometry analysis after staining permeabilised, monocyte-derived DC with a labelled anti-GPC-3 monoclonal antibody detected intracellular staining of GPC-3 only in matured, GPC-3 mRNA transfected DC and not in matured, control DC; we did not detect surface expression of GPC-3 in any DC. The reason for the discrepancy between our findings and those of Wegrowski et al [30] needs further investigation, but they utilised RT-PCR to detect GPC-3 mRNA and Western blot to detect the protein both of which are more sensitive assays than the flow cytometry analysis used in the present study. However, it should be emphasised that there was no evidence of stimulation of GPC-3-specific T cells by control DC in the present study. Murine studies have also provided reassuring data, as DC modified to express GPC-3 were shown to elicit effective antitumor immunity with no evidence of induction of autoimmune injury to liver or other organs [12,13,31].

Mature GPC-3 is modified post-translation into a heparan sulphate proteoglycan [8]. Although the addition of the carbohydrate moiety could potentially mask some and generate other novel B-cell epitopes, it will not interfere with the presentation of MHC class I-restricted epitopes to CD8+ T cells. Previously, it was believed that mature cellular proteins were the main source of antigenic peptides but it is now known that MHC class I peptides originate predominantly from newly synthesised proteins [32], around 30% of which are immediately polyubiquitinylated and subsequently cleaved by the proteasome. The resulting peptides of 8-11 residues in length are then transported into the endoplasmic reticulum, by the transporter associated with antigen presentation (TAP) complex, where they are assembled with MHC class I molecules [33]. Given that newly synthesised GPC-3 protein will be processed by the proteasome before post-translational modification, the carbohydrate moiety will not affect the presentation of peptide epitopes by MHC class I molecules. CD8+ T cell receptors recognise only GPC-3 epitopes expressed in association with MHC class I molecules rather than the intact mature protein at the cell surface.

A second aim of this study was to identify HLA-A2-restricted epitopes derived from GPC-3. When we analyzed the amino acid sequence of human GPC-3, 6 sequences were identified that were predicted both to bind to HLA-A2 and to be processed by the proteasome. We used flow cytometry analysis of T2 cells, which are TAP deficient, to measure the half-life of peptide binding to HLA-A2 and identified 4 peptides with prolonged, high affinity binding for HLA-A2. Of these, GPC-3_522-530 _FLAELAYDL, fulfilled our criteria as a naturally processed, HLA-A2-restricted CTL epitope because: i) it was generated by the MHC class I processing pathway in DC transfected with GPC-3 mRNA, and ii) HLA-A2 positive, monocyte-derived DC loaded with the peptide stimulated proliferation in autologous T cells and generated CTL that lysed HLA-A2 and GPC-3 positive tumour cells. One of the peptides GPC-3_169-177 _ELFDSLFPV predicted to have strong binding to HLA-A2 was found to rapidly dissociate from HLA-A2 in the present study and DC loaded with this peptide did not stimulate autologous T cells in HLA-A2 positive subjects, a finding confirmed by Nishimura and colleagues who found that DC loaded with GPC-3_169-177 _ELFDSLFPV were unable to induce CTL or T cells producing interferon-gamma [34].

Previously, Komori *et al *used HLA-A2.1 transgenic mice to identify HLA-A2 (A*0201)-restricted GPC-3 epitopes but found no evidence that CTL were generated against GPC-3_522-530 _FLAELAYDL in animals immunized with DC pulsed with a mixture of peptides because, after spleen cell harvest, only CD4^- ^T cells stimulated *in vitro *with the peptide GPC-3_144-152 _FVGEFFTDV produced high levels of interferon-γ[31]. These findings suggest that the epitope GPC-3_144-152 _might be immunodominant in this system or, alternatively, CTL reactive to GPC-3_522-530 _may not have been generated in HLA-A2.1 transgenic mice due to differences in the T cell repertoire between mice and humans, resulting in some HLA-A2-restricted epitopes being recognized only by human T cells. Non-dominant epitopes, although having a weaker affinity to MHC, can still induce reactive CTL with cytotoxic activity and thus be applicable for immunotherapy [35]. Indeed, T cells responding to such epitopes are often better represented in the peripheral T cell repertoire because those responding to self-epitopes with strong MHC binding are more likely to be deleted in the thymus during the ontogeny of the immune system [36].

Three additional GPC-3 peptides found to have high affinity binding for HLA-A2 in the present study are thought to be less suitable for further clinical investigation because either T cells from the peripheral T cell repertoire were poorly reactive (peptide 1, GPC-3_229-237 _FLQALNLGI), or the epitopes were not generated by the MHC class I processing pathways in the HCC cell line used as the target cell for the CTL assay (peptide 4, GPC-3_186-194 _GLPDSALDI) or in the DC (peptide 5, GPC-3_222-230 _SLQVTRIFL). Interestingly, CTL generated by DC loaded with peptide 5 effectively lysed HepG2 cells, indicating that it was expressed in association with HLA-A2 on the surface of the tumour cells, possibly reflecting differences in the cleavage of the GPC-3 polypeptide by the constitutive proteasome in the tumour cell line and the immunoproteasome in DC [37]. Variable numbers of CD8+ precursor T cells in the small number of donors tested or less efficient presentation of peptide 5 by the DC, relative to peptide 2, seem unlikely explanations for the findings as two rounds of stimulation by DC loaded with peptide 5 induced high levels of T cell proliferation and functional CTL in all subjects tested.

GPC-3 appears to be an eminently suitable target molecule for HCC immunotherapy because it is a foetal protein [8] that is expressed early in the development of HCC [38] and has been implicated directly in tumour progression. Membrane bound GPC-3 has been postulated to stimulate the growth of HCC by both facilitating the interaction of Wnt with its signalling receptors [39] and enhancing fibroblast growth factor 2 signalling [40]. Activation of the canonical Wnt pathway is a frequent event associated with the malignant transformation of hepatocytes [41], leading to a rise in β-catenin in the nucleus, which in turn regulates transcription factors controlling hepatoma cell growth [42,43]. Knockdown of GPC-3 was found to attenuate fibroblast growth factor 2 binding, a mitogen that promotes HCC cell proliferation and migration by activating downstream protein kinase pathways [40]. In addition, GPC-3 expression stimulates the recruitment of macrophages into HCC, especially macrophages with a phenotype promoting tumour progression and metastasis [44]. Therefore, although the generation of escape mutants due to loss of expression or mutation of a TAA could lead to the failure of immunotherapy, loss of GPC-3 expression by HCC, under the selective pressure of attack by antigen specific T cells, is likely to be mitigated by diminished tumour growth and invasiveness.

## Conclusions

The findings of this study confirm previous reports that electroporation of mRNA encoding a TAA is an efficient method to load human monocyte-derived DC with antigen [45]. GPC-3 mRNA transfected DC generated GPC-3-reactive T cells that were functional, as shown by interferon-gamma production.

This study also identified a peptide, GPC-3_522-530 _FLAELAYDL, that fulfilled criteria as a naturally processed, HLA-A2-restricted CTL epitope. We anticipate that this epitope and the HLA-A2-restricted GPC-3 epitope, GPC3_144-152 _FVGEFFTDV, identified by a previous HLA-A2 transgenic mouse study [31], can be utilized to monitor CTL responses in patients undergoing immunotherapy studies of GPC-3-loaded DC. These studies will determine the probability of successful generation of HLA-A2-restricted CTL reactive to these epitopes in patients with malignancy.

## List of abbreviations

GPC-3: glypican-3; HCC: hepatocellular carcinoma; PBMC: peripheral blood mononuclear cells; DC: dendritic cells; LPS: Lipopolysaccharide; TIL: tumour infiltrating lymphocytes; IL-2: interleukin 2; TAA: tumour-associated antigens; CTL: cytotoxic T lymphocyte; TAP: transporters associated with antigen processing; IL-4: interleukin-4; eGFP: enhanced green fluorescent protein.

## Competing interests

The authors declare that they have no competing interests.

## Authors' contributions

JOB generated the GPC-3 cDNA and inserted it into the mRNA expression vector, carried out the immunoassays, and drafted the manuscript. FF participated in design, coordination of the study, and helped draft the manuscript. PMH conceived the study, designed the mRNA expression vector, helped to perform the statistical analysis and draft the manuscript. All authors read and approved the final manuscript.
